# A Novel Translational Reprogramming of the Insertion Sequence IS1239 by 1-bp Deletion Resulted in Polymorphism of C-Terminal Amino Acid Sequences in the Pathogenic Bacterium *Streptococcus pyogenes*

**DOI:** 10.4014/jmb.2508.08039

**Published:** 2026-05-07

**Authors:** Yun-Juan Bao, Zhong Liang, Zhi-Song Chen, Francis J. Castellino

**Affiliations:** 1 W.M. Keck Center for Transgene Research, University of Notre Dame, Indiana 46556, USA; 2 Department of Chemistry and Biochemistry, University of Notre Dame, Indiana 46556, USA; 3 School of Life Sciences, Hubei University, Wuhan 430062, P. R.China

**Keywords:** *Streptococcus pyogenes*, Insertion sequences, Mobile elements, Evolutionary selection

## Abstract

The insertion sequence family IS1239 has been found to mediate multiple asymmetric rearrangements in the pathogenic bacterium, Group A *Streptococcus pyogenes* (GAS), but not symmetric rearrangements as observed commonly in other bacteria. Sequence characterizations showed that the copies of IS1239 in GAS encode variable C-termini by utilizing flanking sequences downstream the insertion sites. We found that the variability of the C-termini was caused by translational reprogramming from a 1-bp frame-shift deletion at the 3’-terminus of the gene. The translational reprogramming results in extension and polymorphism of the C-terminal sequences, which was only observed in GAS. Selection analyses indicated that the C-terminal region of IS1239 proteins in GAS is under relaxed selection constraints, thus allowing more sequence changes in this region without affecting the transposition activity of this enzyme. We also showed that the insertion of IS1239 and the formation of flexible C-termini exhibit signatures related to evolutionary selection based on three lines of evidence: (i) copies of IS1239 in GAS were inserted in conserved genomic regions compared to those from *Streptococcus pneumoniae*; (ii) copies of IS1239 in GAS are more frequently inserted in the neighboring regions of tRNAs, rRNAs, or ribosomal proteins, but rarely interrupt their normal coding; (iii) the sites predicted to be under relaxed selective constraints in GAS lineage are enriched in regions outside the functional domains and near the 1-bp deletion which induced the polymorphism of C-termini of IS1239 in GAS.

## Introduction

Insertion sequences (IS) are the simplest form of transposable elements. The genes encoded by IS are usually short, of length 700−2500 bp, encoding the enzyme, transposase, that catalyzes the insertion of IS sequences into insertion sites [1]. The IS also contain terminal inverted repeats on both boundaries, mainly involved in transposase binding, cleavage, and strand transfer during the transposition reactions. Most of the IS generate two target direct repeats in the host genomes each flanking the terminal inverted repeats once they are integrated in the host genomes (Fig. 1A). The autonomous transposition activity of the IS has been shown to confer functional advantages and genetic diversity to the host species. Numerous studies have reported the role of IS in promoting acquisition of accessory genes, thus providing novel functions to host genomes, such as antibiotic resistance and/or enhanced virulence. In addition, IS were found to be frequently involved in genome structural rearrangements *via* homologous recombination, yielding other manners of genetic diversification. Specifically, many of the genomic inversions induced by IS, by acting in concert with another copy, are symmetric around the replication axis (X-shape inversions in linear-form genomes), such that the translocated fragment at the two replication forks is equidistant from the replication origin. This kind of symmetric inversion induced by IS are commonly observed in bacterial lineages [2, 3] and have been proposed to be associated with replication-selection pressure [4, 5].

Previously, we identified a unique family of IS, IS1239, responsible for multiple asymmetric rearrangements in the genome of a hypervirulent Group A *Streptococcus pyogenes* (GAS) isolate, M23ND [6]. Four copies of IS1239 were shown to be involved in the rearrangements, resulting in one translocation, one inversed translocation and one inversion (Fig. S1A). All of these rearrangements are asymmetric around the replication axis (Fig. 3 in the study of Ref. 7) [7]. These asymmetric rearrangements are in contrast with the symmetric inversions in many other cases (Fig. S1B and S1C). It intrigued us to characterize the detailed molecular properties of IS1239 from other bacterial species and investigate the molecular evolution of IS1239.

## Materials and Methods

### Identification of Homologous Copies within Genomes and Orthologous Members of IS1239 among Different Bacterial Species

The protein sequences of IS1239 from GAS were used as blast queries against the NCBI bacterial databases of streptococcus species (as of Nov. 2024). For *S. pneumoniae*, only the genomes in the set of Refseq_genome were used. Only the sequences that were of > 60% similarity to the query were considered. One unique hit from *S. pseudopneumoniae* IS7493 with 108 bp missing in the middle of the gene was not included in the analysis. The copies within the same species were classified in the same orthologous group if the similarity is greater than 95%. For each group, the consensus sequence was reconstructed based on multiple alignments using Clustal Omega [8] and used as the representative for that group. The terminal inverted repeats were determined by extracting and aligning the 350-bp flanking sequences upstream and downstream of each copy of IS1239 in the genomes. The direct target repeats were determined by comparison of the paired 40-bp sequences flanking the terminal inverted repeats. The final average length of terminal inverted repeats is ~27 bp, and that of direct repeats is ~14 bp.

### Visualization and Processing of Sequences

The aligned sequences of IS1239 and upstream/downstream sequences were processed using BioEdit (https://thalljiscience.github.io/page2.html) and in-house script. The sequences were visualized using Jalview [9].

### Quantitative Real-Time PCR (qRT-PCR) Analysis

The GAS bacteria were cultured overnight at 37°C in the Todd-Hewitt broth (BD Biosciences, USA) and the cells were collected from colonies grown to mid-log phase (LP; *A*_600_ = 0.5~0.6) and stationary phase (SP; *A*_600_ = 0.9~1.0) followed by digestion with mutanolysin in spheroplasting buffer. Total RNA was extracted using the RNeasy minikit (Qiagen, USA) and treated twice with DNase from the DNA-free kit. The extracted RNA (100 ng) was reverse transcribed to cDNA using the iScript cDNA synthesis kit (Bio-Rad Laboratories, Inc., USA). Three independent extractions of the total RNA were used. Quantitative PCRs were performed with 12 μL of 2.5x SYBR green PCR master mix (Applied Biosystems, USA) and 100 nM forward and reverse primers (Table S1). The gene *plr*/GAPDH (glyceraldhyde-3-phosphate dehydrogenase) was used as a positive control, and a RNA sample without reverse transcriptase was used as a DNA control.

### Evaluation of the Conservation Level of Upstream/Downstream Sequences of IS1239

The 200-bp upstream and downstream sequences were first extracted for all copies of IS1239 from GAS and *S. pneumoniae*. Then the upstream/downstream sequences of each IS1239 copy were aligned to a selected set of genomes of GAS and *S. pneumoniae* (with complete assembly), respectively. A total of 164 GAS genomes and 185 *S. pneumonia* genomes with complete assembly were used for alignment in order to reduce the alignment bias from fragmented genome assembly.

### Phylogenetic Analysis of IS1239

The phylogenetic structure was inferred using the maximum likelihood method implemented in MEGA11 [10]. The best-fit nucleotide substitution model (Tamura 3-parameter model and 4+gamma substitution categories) was selected based on Bayes information criterion (BIC) using Modeltest [11]. The tree was also constructed with RAxML based on the GTRGAMMA model [12]. The tree topologies are consistent between different methods. The ancestor sequences at each node were predicted using PAML with the maximum likelihood method [13-15].

### Calculation of Substitution Rates in Coding Region and Non-Coding Regions in IS1239

The comparison of the synonymous substitution rate (dS) in IS1239 and the nucleotide substitution rate (μ) in the non-coding region at the 5′-terminus of IS1239 were based on the Nei & Gojobori method [16]. The non-coding region at the 5′-terminus of the 1072-bp core domain of IS1239 contains 101 nucleotide positions from 1-101 in the 1072-bp core domain.

## Results

### Sequence Characteristics of IS1239 in GAS and the Polymorphic C-Terminus

We identified 701 copies of IS1239 from 387 GAS genomes including M23ND (Supplemental File 1). M23ND harbors 22 copies, the highest number among the GAS strains. Four copies of IS1239 are involved in asymmetric arrangements in M23ND and others are only direct inserts without inducing genomic arrangements (Fig. S1A). The core domain of IS1239 are highly conserved across GAS genomes and composed of a 100-bp untranslated region (UTR), the N-terminal DNA-binding domain, and the C-terminal integrase catalytic domain (Fig. 1A). As with many other families of insertion sequences, the core region of IS1239 is delimited by a pair of terminal inverted repeats of ~27 bp, which are further bounded by a pair of nearly identical target direct repeats of roughly 13-15 bp (Fig. 1A). The two identical target direct repeats formed in the host genome when the IS were integrated in the genome *via* the catalysis by the transposase encoded by the IS. Interestingly, we found that IS1239 does not contain the termination codon within its core region, but rather generates the stop codon by utilizing the sequences flanking the 3'-end (Fig. 1B and 1C). The position of the stop codon is highly variable among different copies, creating remarkably polymorphic C-termini with a length in the range of 3-171 bp. The flexibility of the C-terminus allows the gene to directly use the *in situ* host sequences to continue its transcription and elongate the C-terminus.

To assess whether the IS1239 copies with flexible C-termini can be expressed, we performed quantitative real time-PCR (qRT-PCR) assays for the IS1239 copies. We amplified six representative IS1239 copies at the C-terminus (Loc1, Loc4, Loc13, Loc17, Loc21, and Loc22) from M23ND with variable lengths of C-termini (18-168bp, Supplemental File 1), including two copies involved in the asymmetric rearrangement in the M23ND genome (Fig. 1D). Five of the six copies showed expression levels comparable to that of two reference genes (*htsA* and *atpB*), which were previously shown to be expressed at a medium level (*htsA*, heme iron transport) and a high level (*atpB*, ATP synthase B chain involved in energy metabolism) in M23ND [17] (Fig. 1E).

### The Polymorphic C-Terminus Is Unique to GAS and Was Induced by an Internal 1-bp Deletion

In order to examine the existence of polymorphic C-termini in orthologs of IS1239 from other streptococcus species, we performed homology search against the NCBI bacterial genome database of streptococcus subset and the insertion sequence database ISfinder [18]. Among streptococcus, 13 distinct species (including GAS) contain IS1239 and the copy number of each species varies between 7-1731 with *S. pneumoniae* harboring the most copies probably due to the largest number of sequenced genomes of this specie (Supplementary File 2). We further classified all the copies to 17 orthologous groups (with group-wise amino acid similarity 65%-95%) (Supplementary File 2). For downstream analysis, we represented each orthologous group using the consensus sequences of the copies within each group. The characteristics of the orthologous sequences of IS1239 are summarized as following in terms of target direct repeats, terminal inverted repeats, and the catalytic motif [19]. Specifically, (i) although the copies of IS1239 were inserted at random locations of the genomes, the insertion site target sequences (or direct repeat sequences) have a preference for AT-rich stretches of average length ~ 14 bp (Fig. 2A-2D). The exceptions were observed for variant 2 and variant 3 from *S. pneumoniae* (*S. pneumoniae* var2 and var3) (Fig. 2C and 2D). The copies within each of these two groups were inserted at identical target site sequences across multiple *S. pneumoniae* genomes (Fig. 2C and 2D); (ii) the terminal inverted repeat sequences of IS1239 are highly conserved across different copies and groups (Fig. 2E), probably related to the binding of the transposase encoded by IS1239, or transposition regulation; (iii) all copies of IS1239 carry a single ORF encoding the DNA-binding motif at the N-terminus and the catalytic domain at the C-terminal half. We identified the DDDE catalytic sites [1,19] and the patterns of surrounding amino acids typical of the family IS1239 (Fig. 2F and Fig. S2). Overall, the sequence characterization shows that we have confidently identified the orthologous sequences of IS1239 in diverse streptococcal species.

We next examined the existence of C-terminal variability among the copies of each ortholog of IS1239. Interestingly, we did not observe the variability in C-terminal sequences in the copies of other members of IS1239, except from *S. dysgalactiae* subsp. *equisimilis*. This species has been proposed to have exchanged the genomic contents extensively with GAS [20,21]. Over 25% of the mobile genetic elements related regions in *S. dysgalactiae* subsp. *equisimilis* including insertion sequences, have been reported to be highly similar to GAS [21], however, it is still unknown whether the conserved regions are induced by heritance from the common ancestor or ongoing genetic transfer.

Multiple sequence alignments of the orthologs of IS1239 revealed that the variability at the C-terminus of IS1239 among the copies in the GAS group is caused by a 1-bp deletion at the 316^th^ codon, resulting in a +1 frame-shift in comparison with other members (Fig. 3). The 1-bp deletion neither occurs at a slippery codon in a heptanucleotide sequence, YYYXXXZ, as previously commonly observed [22-25], nor causes a translational +1 frame-shift *per se* as reported in some virus RNA genes [26], thus showing that the sequences do not encode a second ORF. Rather, this deletion generates polymorphic C-terminal sequences starting from codon-325 by allowing the 3?-end to invade the downstream sequences until encountering the first *in situ* stop codon.

The sequences of *S. pneumoniae* var2 and var3 also contain a 1-bp deletion at the same position of codon-316, but the copies in each of the two groups are inserted at identical target sites across the genomes of *S. pneumoniae* (Fig. 2 and 2D). Accordingly, the copies of *S. pneumoniae* var2 are inserted at around the genomic location of 1.3-1.4 million bp with the same upstream/downstream genes encoding GNAT family Nacetyltransferase and FOF1 ATP synthase subunit C, respectively, while the copies of *S. pneumoniae* var3 are inserted at around the genomic location of 0.8-0.9 million bp with the same upstream/downstream genes encoding VIT family protein and aminotransferase class I/II-fold pyridoxal phosphate-dependent enzyme, respectively (Supplementary File 2). Due to the low variability of the insertion sites, *S. pneumoniae* var2 and var3 do not produce polymorphic C-termini.

### The Insertion Locations of IS1239 Show Preference for Conserved Regions in GAS

Since the utilization of downstream sequences for the C-terminus of IS1239 was only observed in the group of GAS and *S. dysgalactiae* subsp. *equisimilis*, we examined the insertion site preference of the IS1239 copies in GAS genomes. We first extracted the 200-bp sequences upstream and downstream insertion site of each copy of IS1239 from GAS and evaluated the conservation of each 200-bp sequence among GAS genomes (See Methods & Materials). The conservation was compared with that in *S. pneumoniae* (Fig. 4). The data indicates that the copies of IS1239 in GAS are preferably inserted at sites within conserved regions in comparison with those in *S. pneumoniae* (*P* = 1.2 × 10^-6^ for upstream sequences, and *P* = 2.6 × 10^-7^ for downstream sequences; Chi-squared test) (Fig. 4). Next, we examined the functional properties encoded by the upstream and downstream sequences by identifying the immediate neighboring genes of the copies of IS1239 from GAS and compared the neighboring genes of IS1239 from *S. pneumoniae* (Supplementary File 3). It was found that the neighboring regions encoding tRNAs, rRNAs, or ribosomal proteins are the hot-spots for integration of IS1239 in the GAS genomes with 359 spots out of 701 insertion sites, in comparison with 123 spots out of 820 insertion sites in *S. pneumoniae* var1 (*P* < 2.2 × 10^-16^; Chi-squared test).

In order to examine whether the insertion preference for GAS over *S. pneumoniae* var1 is related with differential genetic properties of the two groups of genomes, we compared four genetic factors, *i.e.*, AT contents of the insertion target sequences, GC contents of the whole genomic background, gene density, and proximity to the replication origin between GAS and *S. pneumoniae* var1. We first examined the AT contents of the insertion target site sequences of IS1239 copies in the two groups (Fig. 2A and 2B). Although the average AT contents of the target sequences is different in GAS and *S. pneumoniae* var1 (73.3% vs. 71.6%), the difference has no statistic significance (*P* = 0.5755, Wilcoxon rank sum test). It indicates that IS1239 prefer to insert at sites with specific level of AT contents regardless of neighboring genes. Secondly, we calculated the genome-wide GC contents of the two groups of genomes containing IS1239 (Fig. S3A). It shows that the GC content is significantly different between GAS and *S. pneumoniae* var1 (*P* < 10^−15^, Wilcoxon rank sum test) indicating distinct genetic background of the two species. Thirdly, we estimated the average gene density of the two groups by calculating number of genes per kilobases for each complete genome of GAS and *S. pneumoniae* var1 containing IS1239 (Fig. S3B). GAS has an average of 1.002 genes per kilobases (0.967-1.04) and *S. pneumoniae* var1 has 1.035 (1.0-1.065). The distribution of gene density in the two groups is significantly different (*P* < 10^-15^, Wilcoxon rank sum test). Lastly, we compared the proximity of IS1239 copies to replication axis between the two groups by calculating the distance proportion of the locations of IS1239 copies to the replication origin of the genomes (only completely assembled genomes were considered) (Fig. S3C). The IS1239 copies do not show strand bias in GAS or *S. pneumoniae* var1 and no significant difference between the two groups (*P* = 0.303, Wilcoxon rank sum test). The comparison of multiple genetic factors shows that GC contents and gene density are significantly different between GAS or *S. pneumoniae* var1 and might be two factors contributing to the insertion preference of IS1239 for GAS over *S. pneumoniae* var1.

In our studied data, the flexible C-termini and frame-shifted IS1239 are exclusively observed in the group of GAS and *S. dysgalactiae* subsp. *equisimilis* and the asymmetric recombination facilitated by IS1239 is only detected in GAS, but not any other species. Although the 1-bp frame-shift deletion is also observed in *S. dysgalactiae* subsp. *equisimilis*., IS1239 has not been found to induce asymmetric recombination in this species.

### Evolutionary Implication from the Phylogenetic Relationship of IS1239 and the Selection Pressures on IS1239 in the GAS Lineage (Clade II)

To investigate the evolutionary insights and potential selection action, we constructed the phylogenetic tree of the IS1239 orthologs. The inclusion of two members of IS1239 (from *S. anginosus* and *S. iniae*) prevents us from building a robust tree (Fig. S4A and S4B), therefore, we excluded those two sequences from analysis. The tree built from the remaining members identifies three main lineages (designated as clade I-III) diverging from a common ancestor (Figs. 5A and Fig. S4C). Notably, the long branch leading to GAS and *S. dysgalactiae* subsp. *equisimilis* in Clade II indicates that this clade has evolved independently from other lineages for a long period of time. Two other members of IS1239, *i.e.*, *S. pneumoniae* var2 and var3, with the identical 1-bp deletion placed in a different lineage (clade III), indicating distinct evolutional path of these two variants from that of GAS.

The independent evolution and long branch leading to the GAS lineage (Clade II) prompted us to test the occurrence of differential selection acting on IS1239 from different lineages during evolution. We tested this hypothesis by measuring the rates of non-synonymous (dN) and synonymous substitutions (dS), and calculating their relative rates (ω = dN/dS), in the sequences of IS1239 assuming homogenous substitutions rate among all sites of IS1239. In the neutral evolution model, the rate of non-synonymous substitutions is equal to that of synonymous substitutions giving rise to ω ~ 1. Any significant deviation of ω from 1 indicates purifying selection (ω < 1) or positive selection (ω > 1). Under the assumption of uniform substitution rates among all sites in the gene of IS1239, we calculated dN/dS for each branch in the phylogeny tree [16]. This analysis shows that the gene IS1239, as a whole, has been mainly subject to purifying selection during its evolution (Fig. 5B). There is no significant signal of positive selection detected that acted on the GAS lineage.

We next tested for the presence of potential selection at specific sites of the gene IS1239 in the GAS lineage using the branch-site models implemented in the software PAML [15, 27, 28]. The likelihood ratio test (LRT) shows that a small proportion of sites among the sequences of IS1239 have a significantly higher ω value in the GAS lineage (ω_G_ = 2.64; *P* = 0.003) compared to a model with uniform values of ω (Model 2a in [29]) (Table 1). The elevated value of ω_G_ is only marginally significant when compared with a null model of ω_G_ = 1. This indicates that the lineage of GAS has been subject to positive selection or relaxed selective constraints at a small proportion of sites relative to all other lineages.

To distinguish between the two possibilities, we further examined the differences in the values of ω among the three clades of the tree, based on a rooted tree, by reconstructing the most likely ancestral sequences at each node of the tree using the maximum likelihood method [13-15]. Three tests were constructed allowing for diverged selection pressures between Clade II and Clade I, Clade III, or both Clade I and Clade III (Table 1). In each case, the estimates of the ratio of non-synonymous over synonymous substitutions for the GAS lineage were significantly elevated compared with the ratios for Clade I or Clade II, albeit less than 1 (ω_G_ = 0.80; ω_I_ =0.495; ω_III_ =0.049; p ≤ 0.0006). The tests support the latter possibility that relaxed selective constraints have acted on the GAS lineage. However, the elevated value of ω could also arise from increased constraints against synonymous substitutions in the GAS lineage. We evaluated this possibility by comparing the ratio dS/μ between synonymous substitution rate, dS, in IS1239 and the nucleotide substitution rate, μ, in the non-coding region at the 5′-terminus of IS1239, assuming the neutral substitution rate in non-coding regions. We obtained a ~10-fold higher dS/μ for the GAS lineage compared with that for Clade I/Clade III (with the median 2.1 vs. 0.26). Therefore, it is highly unlikely that the increased ω value is induced by the enhanced constraints against synonymous substitutions, providing further evidence for relaxed selective constraints acting on a proportion of the sites in IS1239 from the GAS lineage.

We next sought to determine the sites under relaxed selective constraints in the GAS lineage. Because the reliability of the site identifications using sophisticated maximum likelihood methods greatly depend on the accuracy of tree topology, root reconstruction, and sufficient sampling capacity in the lineages (the total sampling in the GAS lineage is very low), we identified the sites under potential relaxed selective constraints in the GAS lineage by tabulating only the radical nucleotide substitutions (those substitutions causing amino acid changes with different chemical properties in terms of charge and hydrophobicity) unique to the GAS lineage (Supplementary File 4).

To examine the functional implication of the sites undergoing relaxed selective constraints, we mapped the target sites to the core domain of IS1239, and found that the target sites are enriched in regions outside the two functional domains (*p* ~ 0.001; binomial test) (Fig. 6). Interestingly, six of the target sites are concentrated at the 3′-terminus near the 1-bp deletion that is specific to the GAS lineage. The clustering of the target sites suggests that the region surrounding the frame-shift deletion shows signals of evolving under relaxed selections such that radical alteration of the amino acids in this region is allowed without affecting the transposition activity of the protein required under diverse host circumstances.

### Test of Selections in Other Clades: Clade I and Clade III

Next we tested the selection properties and their heterogeneity among Clade I and Clade III. In order to reduce the complexity induced by the interaction between Clade II and the other two clades, we temporarily removed the sequences from Clade II and derived a robust bifurcating tree for Clade I and Clade III (Fig. S5). Two LRT tests were conducted with either of the two branches evolving as the foreground branch in the branch-site model A [27]. We obtained a significant positive selection at a fraction of sites at the branches leading to Clade I and Clade III. The two tests generated identical target sites under positive selection due to the undirectional tree of the two lineages. In order to explicitly identify the sites under positive selection for each of the two clades, we constructed two additional LRT tests using a close relative of IS1239, *i.e.*, IS1161, as an outgroup (Table 1). The tests provide further evidence for the positive selection in both Clade I (ω_I_ =21.6; *P* = 0.0001) and Clade III (ω_III_ =12.0; *P* = 0.001) at a small proportion of sites. At a posterior probability threshold of ≥ 80% from the Bayes empirical test implemented in PAML [30], we obtained seven and five sites evolving under positive selection in Clade I and Clade III, respectively (Supplementary File 4). All but one of those sites are distributed within the two functional domains of IS1239, *i.e.*, the DNA-binding domain and the integrase catalytic domain (Fig. 6). This distribution profile is in contrast with the location of the sites under relaxed selective constraints in the GAS lineage, which are clustered outside the functional domains.

## Discussion

We have characterized a unique insertion sequence, IS1239, in GAS genomes, which encodes variable C-termini by utilizing diverse flanking sequences downstream the insertion sites. We showed that the variability of the C-termini was caused by a 1-bp frame-shift deletion at the C-terminus of the gene. This 1-bp frame-shift deletion results in extension and polymorphism of their C-terminal sequences. By comparison with the orthologs of IS1239 in other streptococcus species, we found that the polymorphism of the C-termini is species/lineage specific and only identified in the lineage comprising GAS genomes and its close recombining relative *S. dysgalactiae* subsp. *equisimilis*. Although the 1-bp deletion also occurred in two other members of IS1239, *i.e.* *S. pneumoniae* var2 and var3, they do not produce polymorphism at the C-termini. Actually, IS1239 copies of *S. pneumoniae* var2 or var3 were inserted at identical direct repeat sequences (as shown in Fig. 2C and 2D). We propose that IS1239 copies from each of these two groups probably were inherited vertically from a common ancestor.

The use of flanking DNA sequences to provide the C-terminal termination signal was also observed in other IS elements, such as IS870 from *Agrobacterium vitis* [31], ISMt1 from *Mycobacterium tuberculosis* [32], IS1547 from *Mycobacterium tuberculosis* [33], and IS1110 from *Mycobacterium avium* [34]. However, the former two transposable elements appear to insert into a specific 5′-CTAG-3′ target site and produce the stop codon within the target sequence “TAG”, while the latter two have limited copy numbers to assess the variability of the C-terminus. Therefore, this flexible C-terminal coding of IS1239 induced by translational reprogramming up to now seems a unique property of GAS and its close relative *S. dysgalactiae* subsp. *equisimilis*. This flexibility can provide important implications in genetic engineering. For instance, it will make it possible to mutate internal genes or insert foreign genes in a genome-wide scale using transposon mutagenesis (loss-of-function) or reverse transposon mutagenesis (gain-of-function) [35].

It was noticed that the copy numbers of IS1239 in different strains of GAS are highly variable. While the integration of IS elements into bacterial genomes is closely related with the matching between their genetic background (*i.e.*, integration site sequences, genomic structure, and co-adaptation), the actual occurrence of integration events are very occasional depending on multiple external environmental factors (such as the number of bacterium colonies, the number of IS copies, and host conditions), especially for a human pathogen like GAS. We noticed that even for the strains with the same *emm* type, the IS1239 copy numbers varied in a wide range. For instance, the M18 strain MGAS8232 contains 15 copies, while M18 M18BO-RO and M18 CPost only contain one copy (see more details in Supplementary File 1).

The reconstruction of phylogenetic history of distinct members of IS1239 indicates that this IS family has diverged into multiple lineages (Clade I-III) and the GAS Clade II appears to have evolved independently for long time based on the long branch length leading to this lineage. Further selection tests reveal relaxed selection constraints acting on the branch leading to the GAS lineage at a small fraction of sites although not at the whole branch. These target sites are enriched in the regions outside the functional domains of IS1239, including the C-terminus near the position of the 1-bp deletion. Although the selection tests based on the relative rates of non-synonymous and synonymous substitution rate is not fit for testing of insertions/deletions, we nonetheless indirectly showed that the C-terminal region of IS1239 shows evidence of relaxed functional constraints thus allowing for more amino acid changes/additions in this region without affecting the transposition activity of the enzyme. With these relaxed functional constraints, we argue that the formation of flexible C-termini of IS1239 is an IS-host co-adaptive consequence and might have been evolutionary selected based on three lines of evidences. Firstly, the copies of IS1239 in GAS were preferentially inserted in conserved genomic regions compared to those from *S. pneumoniae*. Secondly, the copies of IS1239 in GAS were more frequently inserted in the neighboring regions encoding tRNAs, rRNAs, or ribosomal proteins, but rarely interrupting their normal coding (but perhaps affecting their regulation). The preference of IS1239 integration for locations near tRNAs and rRNAs might be a co-adaption consequence between IS1239 and GAS genomes. The redundancy of tRNAs or rRNAs in bacterial genomes [36] may facilitate these genes less susceptible to interruption. However, why IS1239 copies were integrated near tRNAs or rRNAs more frequently in GAS than in other species like *S. pneumoniae* is still unknown and further investigation is warranted. Lastly, the sites predicted to be under relaxed selective constraints in the GAS lineage are enriched in regions outside the functional domains and near the 1-bp deletion which induced the polymorphism of C-termini of IS1239 in GAS.

In addition to the predicted relaxed functional constraints acting on the C-termini of IS1239 in GAS genomes, we also identified significant positive selection at a fraction of sites at the branches leading to the other two clades, Clade I and Clade III. The sites predicted to be under positive selection in these two clades are concentrated in the functional domains of IS1239, in contrast to the distribution of the sites under relaxed functional constraints in GAS. These data point to a scenario of multi-level selections acting on different lineages or regions of IS1239 as a consequence of evolutionary adaptation. In the case of insertion sequences, the adaptation may involve multiple synergistic factors, such as translational regulation, transcriptional regulation, host interaction, and host phylogeny.

## Supplemental Materials

Supplementary data for this paper are available on-line only at http://jmb.or.kr.



## Figures and Tables

**Fig. 1:**
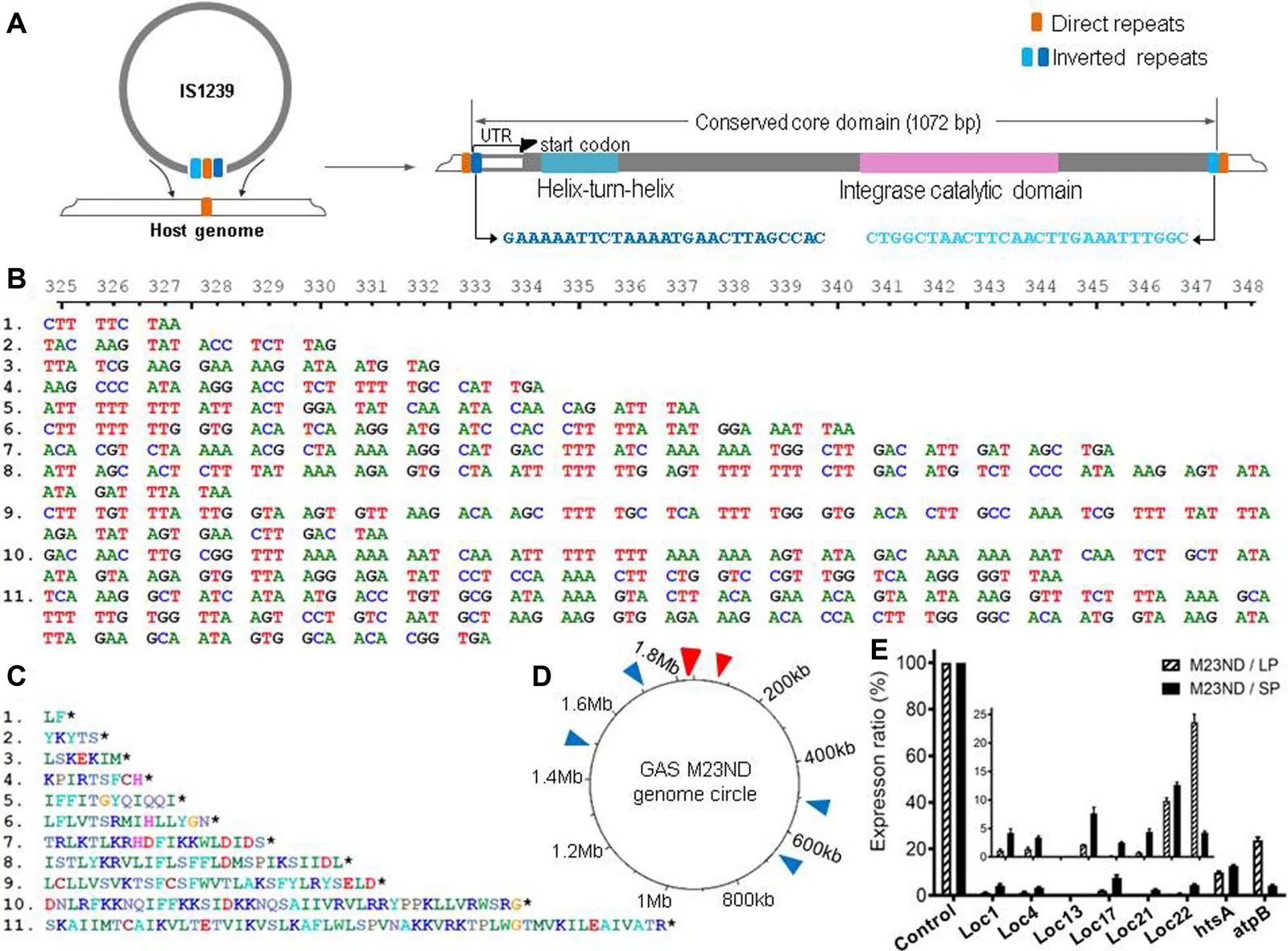
Flexible termini of IS1239 in GAS genomes. (**A**) Illustration of the domain structure of the core region of IS1239 and its flanking region after integrated in the host genome. The core region of IS1239 is composed of a 100-bp UTR region, and the DNA-binding domain and integrase catalytic domain. The core region of IS1239 is delimited by two terminal inverted repeats of ~27 bp, which are further bounded by two nearly identical direct target repeats of roughly 13-15 bp. The inverted repeat sequences are highly conserved in GAS genomes and the consensus sequences are shown in colored texts. However, the C-terminal sequences extend beyond the core region of IS1239 and become highly variable starting from codon-325. (**B**) The C-terminal sequences of IS1239 starting from codon-325. Ten representative IS1239 sequences from the 701 copies with different lengths are shown. (**C**) The translated amino acid sequences of (B) are shown accordingly. The termination codon is shown as “*”. (**D**) Illustration of the six selected copies of IS1239 from M23ND used for qRT-PCR assay. The genomic locations of the copies are indicated in triangle with red indicating the copies involved in asymmetric rearrangement in the M23ND genome. (**E**) The expression levels of the six IS1239 copies from M23ND determined by qRT-PCR at different growth stages (LP: mid-log phase; SP: stationary phase). The expression values were normalized to the control gene *plr*/GAPDH, which has been known to be expressed at a stable level across the growth phases. Two additional genes *htsA* and *atpB* were used as references, which were known to be expressed at a medium level (*htsA*) and a high level (*atpB*) in M23ND.

**Fig. 2:**
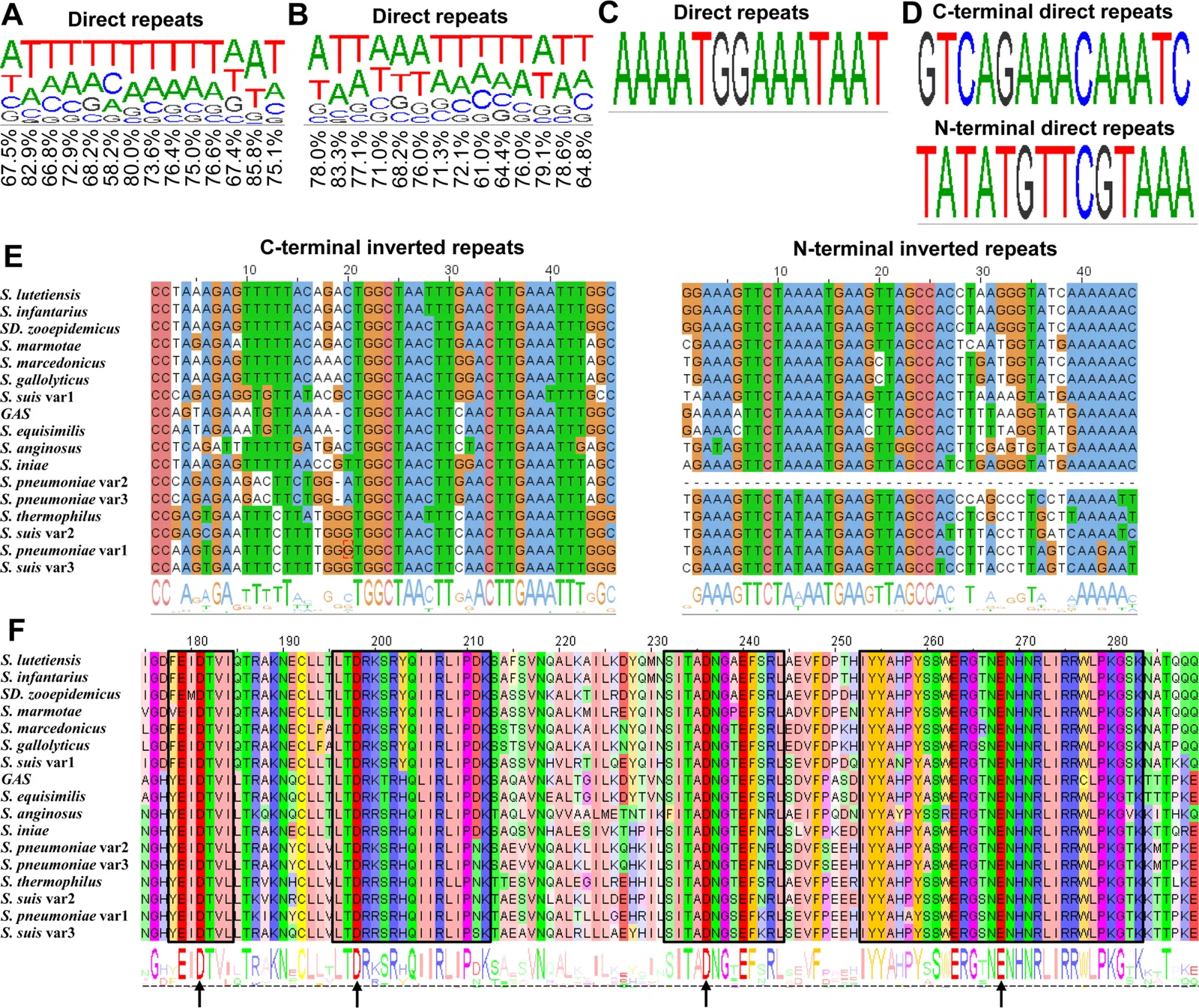
Sequences characteristics of IS1239 from diverse streptococcal species. (**A-B**) The copies of IS1239 were inserted at target sites with AT-rich sequences in GAS (A) and the variant 1 of *S. pneumoniae* (**B**). (**C-D**) The copies of *S. pneumoniae* var2 (**C**) and var3 (**D**) were inserted at identical direct repeat sequences across the genomes. Sequence logos of direct repeat sequences at target sites are shown. The AT-content at each site of direct repeats is shown below the logo by summing over different insertion sites in each orthologous group. The direct repeat sequences are different between the N-terminus and C-terminus for *S. pneumoniae* var3. (**E**) Terminal inverted repeats of all members of IS1239 except from *S. pneumoniae* var2, where the inverted repeats have been lost at the N-terminal side. The sequences are highly conserved across different copies and groups. The consensus sequences are shown at the bottom. (**F**) Amino acid alignment and consensus sequences of the catalytic domain of IS1239 from 17 orthologous groups. The DDDE catalytic sites are identified and indicated with arrows, and the conserved regions surrounding each residue are in frame. The residues are colored using the Zappo color scheme in Jalview based on the physical chemical properties of amino acids: ILVAM (aliphatic/hydrophobic) in pink; FWY (Aromatic) in orange; KRH (positive) in blue; DE (negative) in red; STNQ (hydrophilic) in green; PG (small/flexible) in magenta; C (cysteine) in yellow. The alignment of the full-length IS1239 from 17 orthologous group is presented in Fig. S2.

**Fig. 3:**
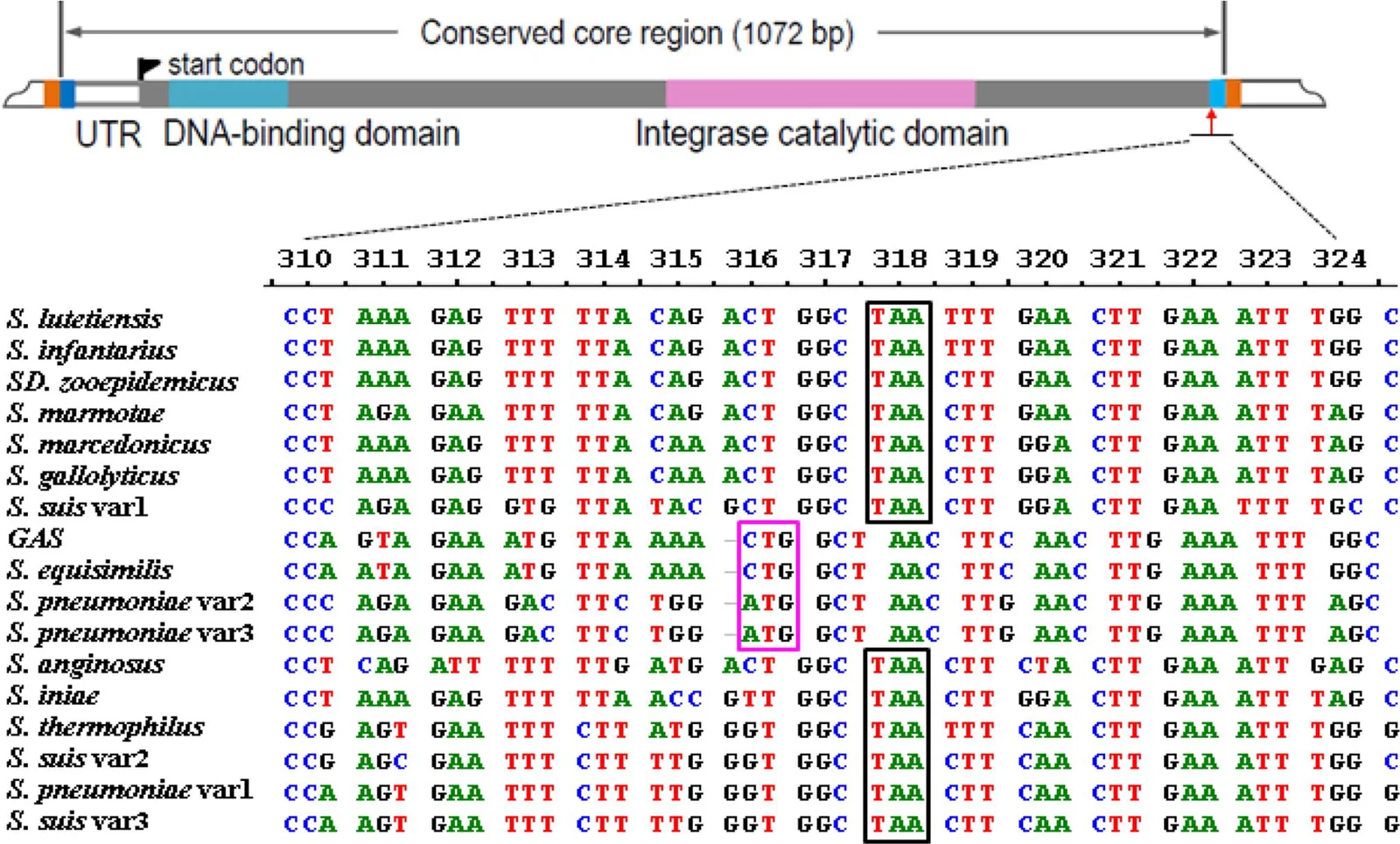
Multiple sequence comparison of orthologous members of IS1239 at the C-termini. The variability of the sequences at the C-termini in the GAS group is induced from a 1-bp deletion at the codon-316, resulting in a +1 frame-shift in comparison with other members. The domain structure of IS1239 at the top is the same as in Fig. 1, and shown as a reference here. The red arrow at the C-terminus indicates the position of the deletion, and the sequences upstream and downstream the deletion are shown in the alignment for all members of IS1239. *S. pneumoniae* var2 and var3 also contain a 1-bp deletion at the same position, but do not produce polymorphisms at the C-termini.

**Fig. 4:**
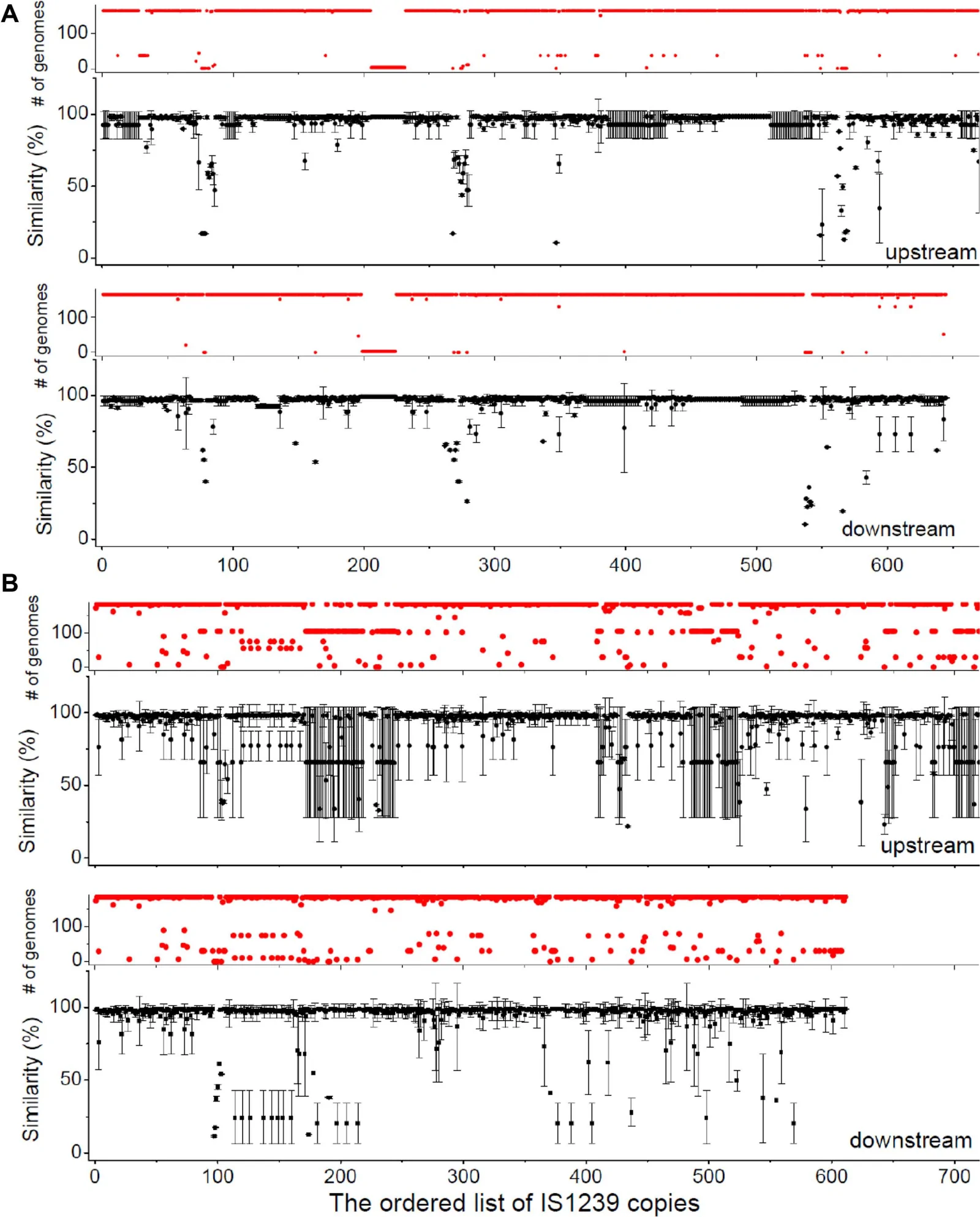
Evaluation of the conservation of 200-bp sequences upstream and downstream the insertion sites of IS1239 copies from GAS (**A**) and *S. pneumoniae* var1 (**B**). Lower panel: the similarities obtained by aligning the 200-bp upstream/downstream sequences of each IS1239 copy to the genomes of GAS (**A**) or *S. pneumoniae* (**B**). The bar indicates the standard deviation of the similarities between each 200-bp sequence fragment with the genomes of GAS or *S. pneumoniae*. Upper panel: the number of genomes which can be aligned with each fragment of upstream/downstream sequences at a similarity > 60%. The upstream/downstream sequence fragment was defined as highly conserved if more than 90% of the genomes of GAS or *S. pneumoniae* can be aligned with the fragment at a similarity > 60%. To reduce the bias from fragmented genomes which were not assembled completely, we only used the completely assembled genomes for the alignment. The difference in the insertion preference of IS1239 between GAS and *S. pneumoniae* was assessed using Chi-squared test by comparing the number of IS1239 copies inserted at sites of high conserved upstream/downstream sequences.

**Fig. 5:**
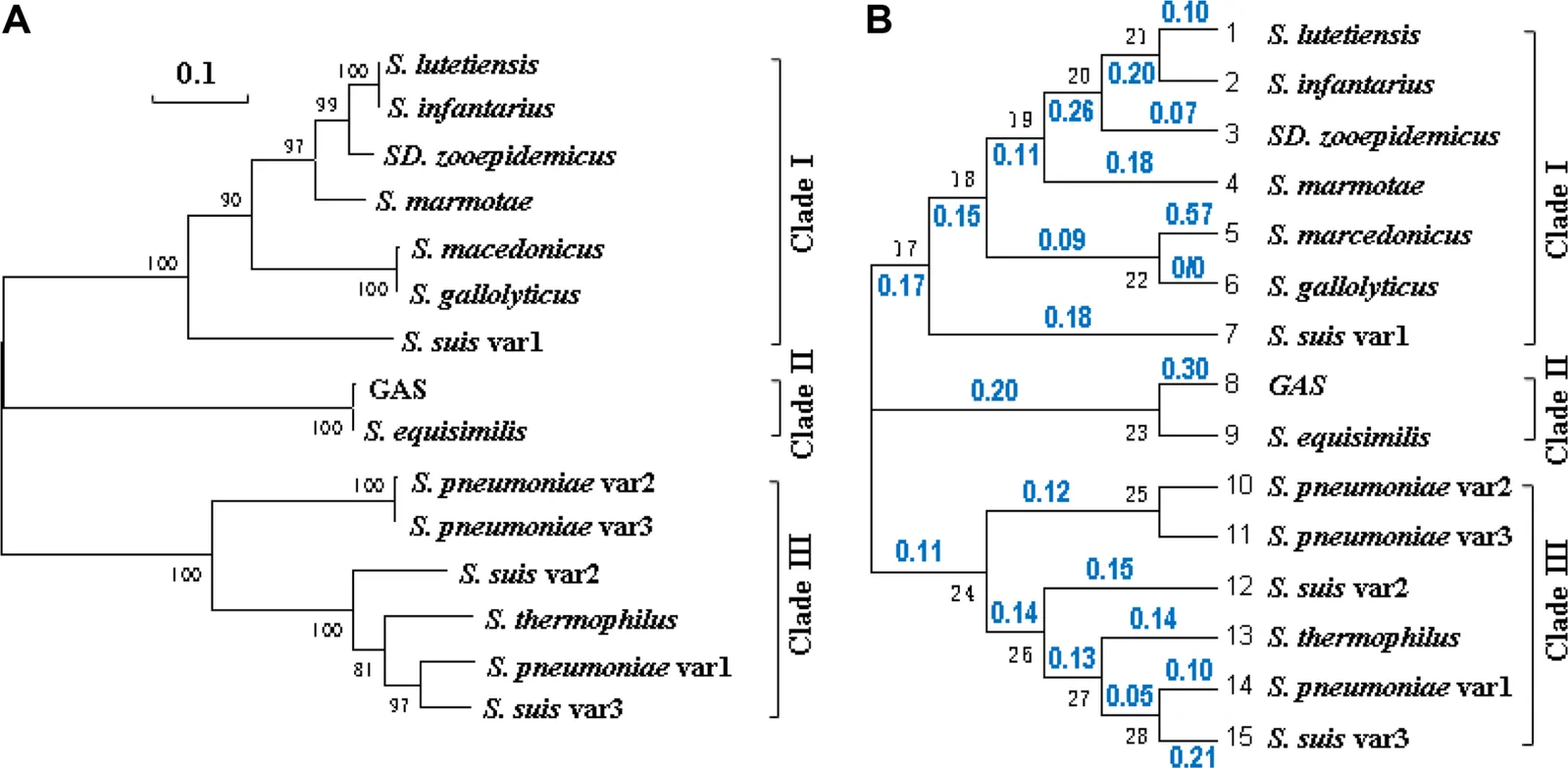
Phylogenetic tree and ratio of non-synonymous to synonymous substitution rates for sequences of IS1239 (excluding two orthologs from *S. anginosus* and *S. iniae*). The tree was built using the maximum likelihood method with Tamura 3-parameter model and 4+gamma substitution categories using the consensus sequences of IS1239 for each group or species. (**A**) The tree branches are shown in scale indicating the number of substitutions per site for each branch. The support values are shown on the branches with 1000 bootstraps. (**B**) The ratio of non-synonymous to synonymous substitutions (ω = dN/dS) were calculated for each branch and shown on the branches in blue. The values are not shown on branches where ω is not accessible. The tree topology is the same as in (**A**) but the branches are not in scale in order for better presentation of ω for each branch.

**Fig. 6:**
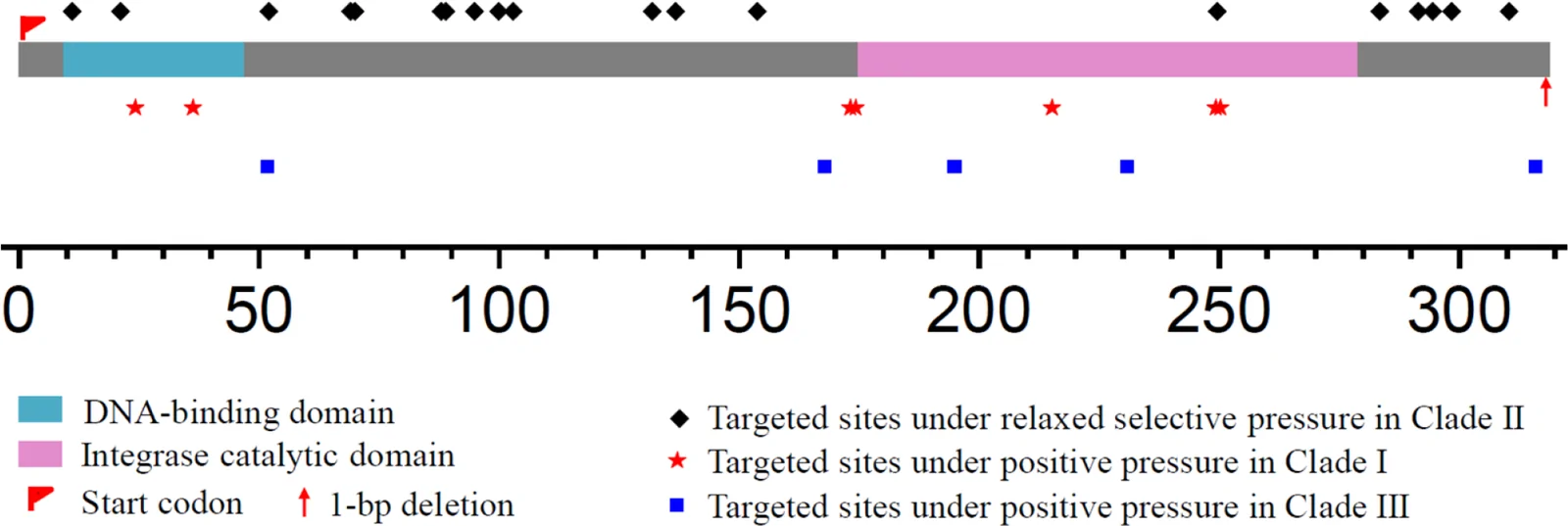
Distribution of target sites under different selections. The sites predicted to be under relaxed selective constraints in the GAS lineage (black diamonds) are enriched in regions outside the two functional domains (*i.e.*, the grey region in the IS1239). The codon positions of the sites in IS1239 are: 11, 21, 52, 69, 70, 88, 89, 95, 100, 103, 132, 137, 154, 250, 284, 292, 295, 299, and 311. Seven (codon positions at 52, 168, 195, 231, and 316) and five sites (codon positions at 24, 36, 173, 174, 215, 249, and 250) were predicted to be under positive selection in Clade I (red stars) and Clade III (blue squares), respectively. Nine of these 12 sites under positive selection in Clade I or Clade III are distributed within the two functional domains, *i.e.*, DNA-binding domain and integrase catalytic domain. Only sites with radical substitutions are presented. The details of the sites presented in this figure can be obtained from Online datasets Supplementary File 4.

**Table 1:** Tests of selections in the three clades of IS1239.

Alternative hypothesis	Null hypothesis	d.f.^1^	*p*-value	Estimates of ω values	Proportion^2^
LRTs of elevated ω in the GAS lineage					
ω_G_ ≠ ω0	ω_G_ = ω0 (M2a)	2	0.0032	ω_G_ = 2.64	*f*_G_ = 0.047
ω_G_ > 1	ω_G_ = 1	1	0.099		
LRTs of variable ω in the three clades					
ω0 ≠ ω_G_ ≠ ω_III_	ω0 = ω_G_ = ω_III_ (M3)	2	0.00015	ω_G_ = 0.803, ω_III_ = 0.049	*f*_G_ = 0.11
ω0 ≠ ω_G_ ≠ ω_I_	ω0 = ω_G_ = ω_I_ (M3)	2	0.00015	ω_G_ = 0.803, ω_I_ = 0.495	*f*_G_ = 0.11
ω0 ≠ ω_G_ ≠ ω_III_ ≠ ω_I_	ω0 = ω_G_ = ω_III_ = ω_I_ (M3)	3	0.00054	ω_G_ = 0.803, ω_III_ = 0.049, ω_I_ = 0.495	*f*_G_ = 0.11
LRTs of divergent ω between Clade I and Clade III					
ω0 ≠ ω_III_ > 1	ω_III_ = 1	1	0.000066	ω_III_ = 126	*f*_III_ = 0.073
ω0 ≠ ω_I_ > 1	ω_I_ = 1	1	0.000066	ω_I_ = 93	*f*_I_ = 0.073
LRTs of divergent ω between Clade I and Clade III with IS1161 as an outgroup					
ω0 ≠ ω_III_ > 1	ω_III_ = 1	1	0.0014	ω_III_ = 12.0	*f*_III_ = 0.007
ω0 ≠ ω_I_ > 1	ω_I_ = 1	1	0.0001	ω_I_ = 21.6	*f*_I_ = 0.007

1. Degree of freedom.

2. Proportions of sites with **ω** values in the alternative hypothesis.
