# Supplementary material for: A Novel Translational Reprogramming of the Insertion Sequence IS1239 by 1-bp Deletion Resulted in Polymorphism of C-Terminal Amino Acid Sequences in the Pathogenic Bacterium Streptococcus pyogenes
Source: J Microbiol Biotechnol. 2026 May 7;36:e2508039. doi: 10.4014/jmb.2508.08039 (PMC13630536; doi:10.4014/jmb.2508.08039)
Supplement: Supplementary file 1 [file jmb-36-e2508039-supple.zip › jmb-36-e2508039-supple.pdf]

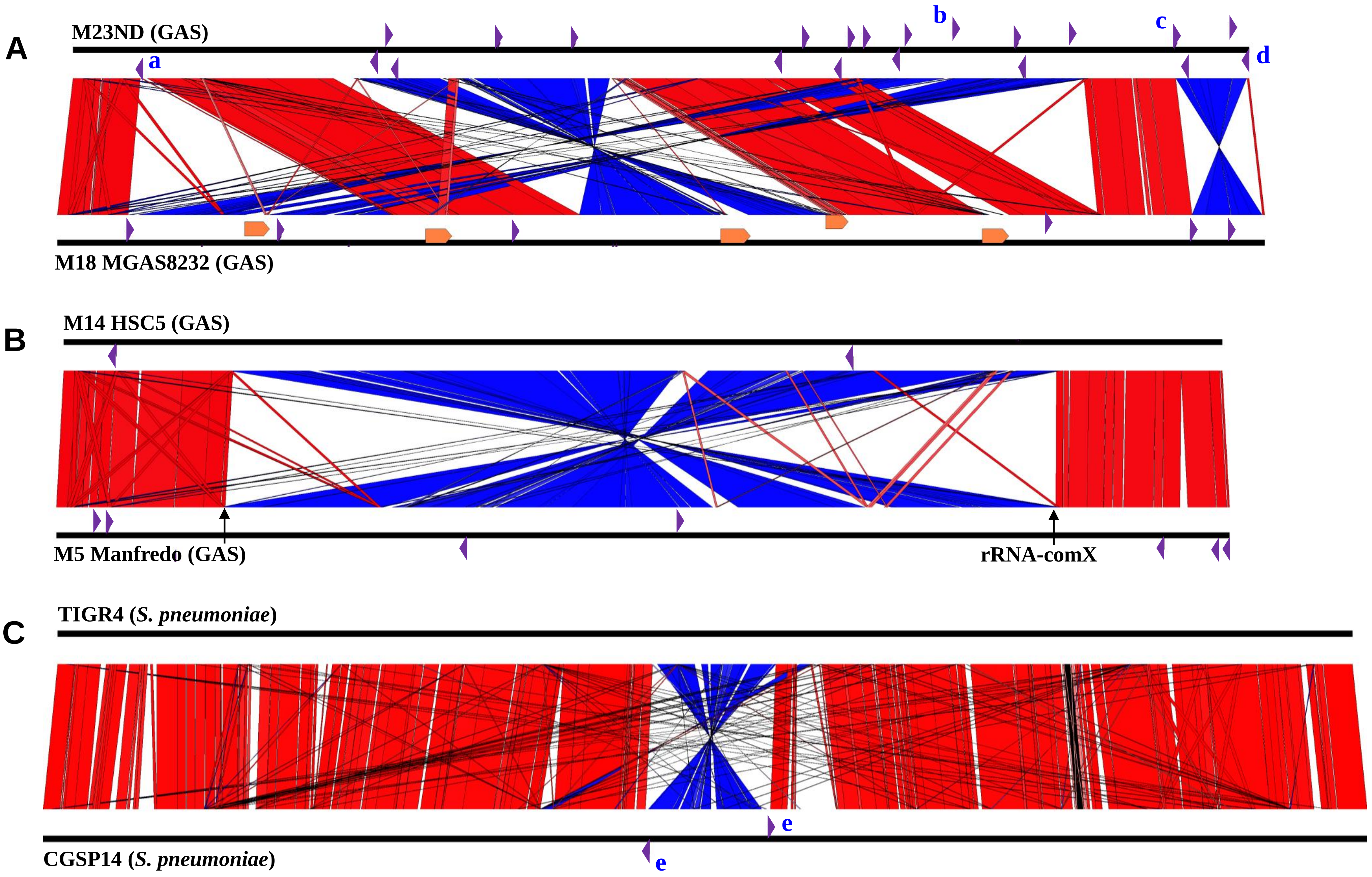
A
M23ND (GAS)
a
b
c
d
M18 MGAS8232 (GAS)
B
M14 HSC5 (GAS)
M5 Manfredo (GAS)
rRNA-comX
C
TIGR4 (S. pneumoniae)
e
e
CGSP14 (S. pneumoniae)

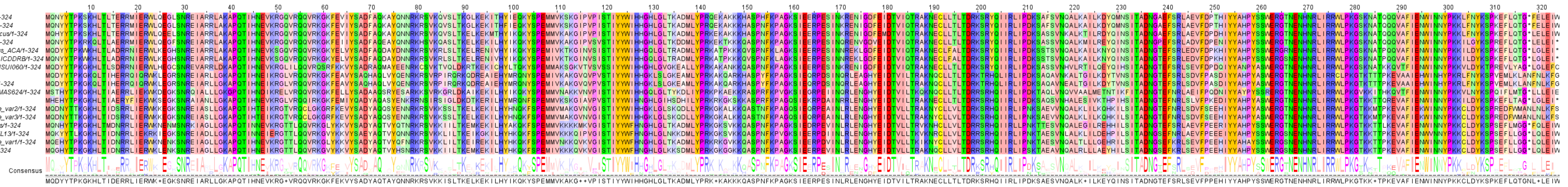

FigureS2_DDE_domain

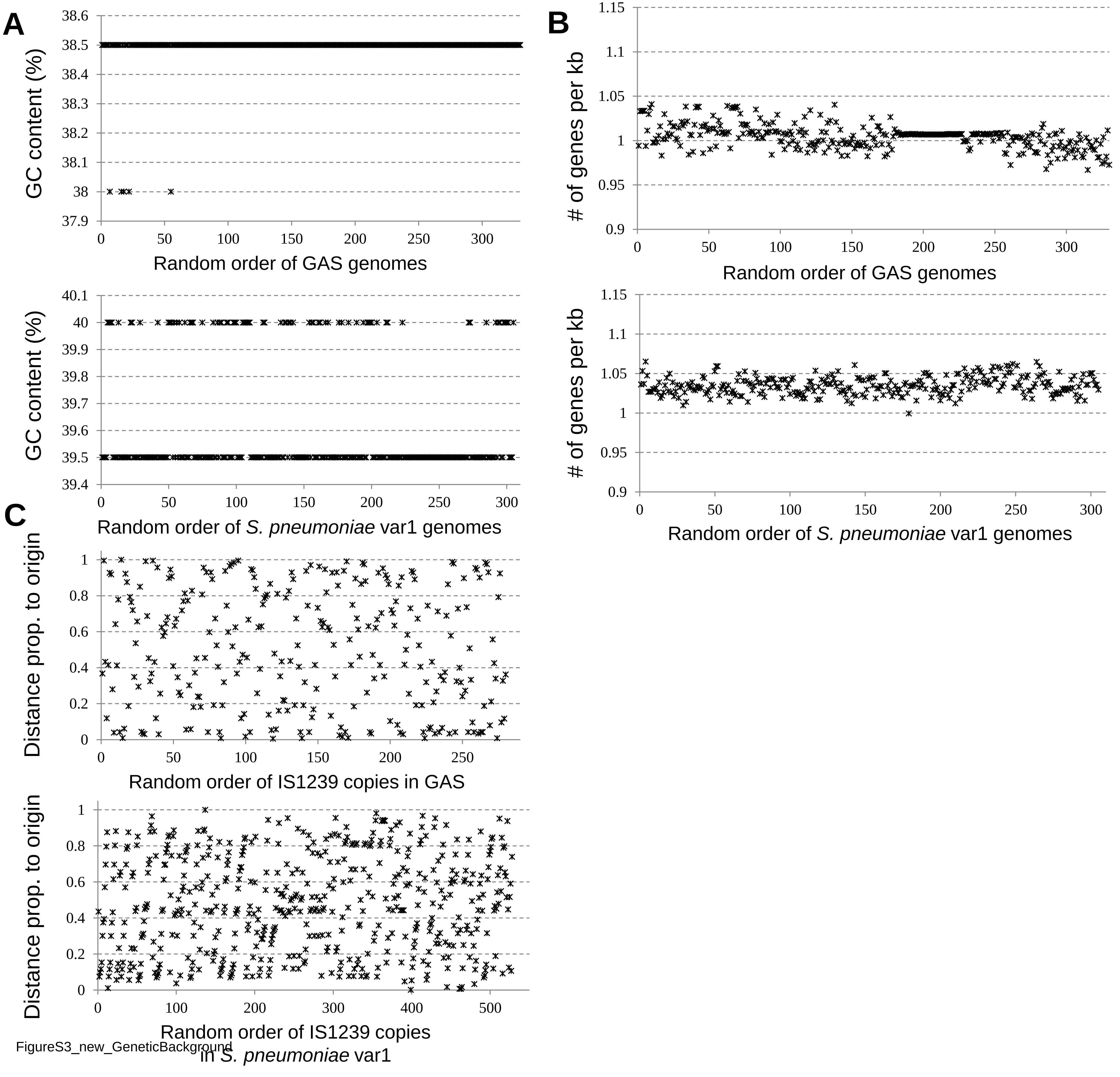
A
GC content (%)
Random order of GAS genomes
GC content (%)
Random order of *S. pneumoniae* var1 genomes
B
# of genes per kb
Random order of GAS genomes
# of genes per kb
Random order of *S. pneumoniae* var1 genomes
C
Distance prop. to origin
Random order of IS1239 copies in GAS
Distance prop. to origin
Random order of IS1239 copies in *S. pneumoniae* var1

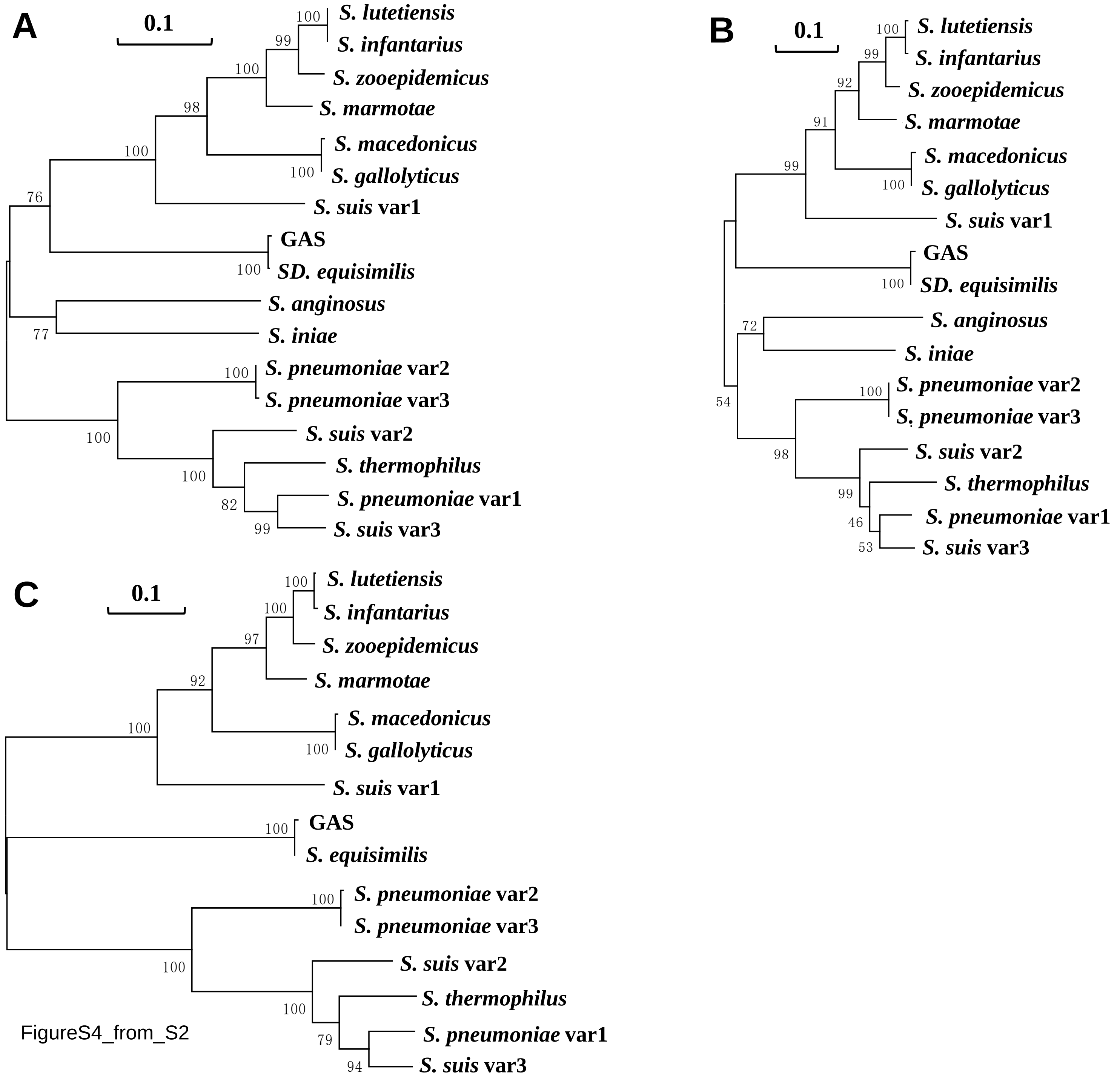
A
0.1
S. lutetiensis
S. infantarius
S. zooepidemicus
S. marmotae
S. macedonicus
S. gallolyticus
S. suis var1
GAS
SD. equisimilis
S. anginosus
S. iniae
S. pneumoniae var2
S. pneumoniae var3
S. suis var2
S. thermophilus
S. pneumoniae var1
S. suis var3
100
99
100
98
100
100
76
100
77
100
100
100
82
99
B
0.1
S. lutetiensis
S. infantarius
S. zooepidemicus
S. marmotae
S. macedonicus
S. gallolyticus
S. suis var1
GAS
SD. equisimilis
S. anginosus
S. iniae
S. pneumoniae var2
S. pneumoniae var3
S. suis var2
S. thermophilus
S. pneumoniae var1
S. suis var3
100
99
92
91
99
100
100
72
54
100
98
99
46
53
C
0.1
S. lutetiensis
S. infantarius
S. zooepidemicus
S. marmotae
S. macedonicus
S. gallolyticus
S. suis var1
GAS
S. equisimilis
S. pneumoniae var2
S. pneumoniae var3
S. suis var2
S. thermophilus
S. pneumoniae var1
S. suis var3
100
100
97
92
100
100
100
100
100
100
79
94

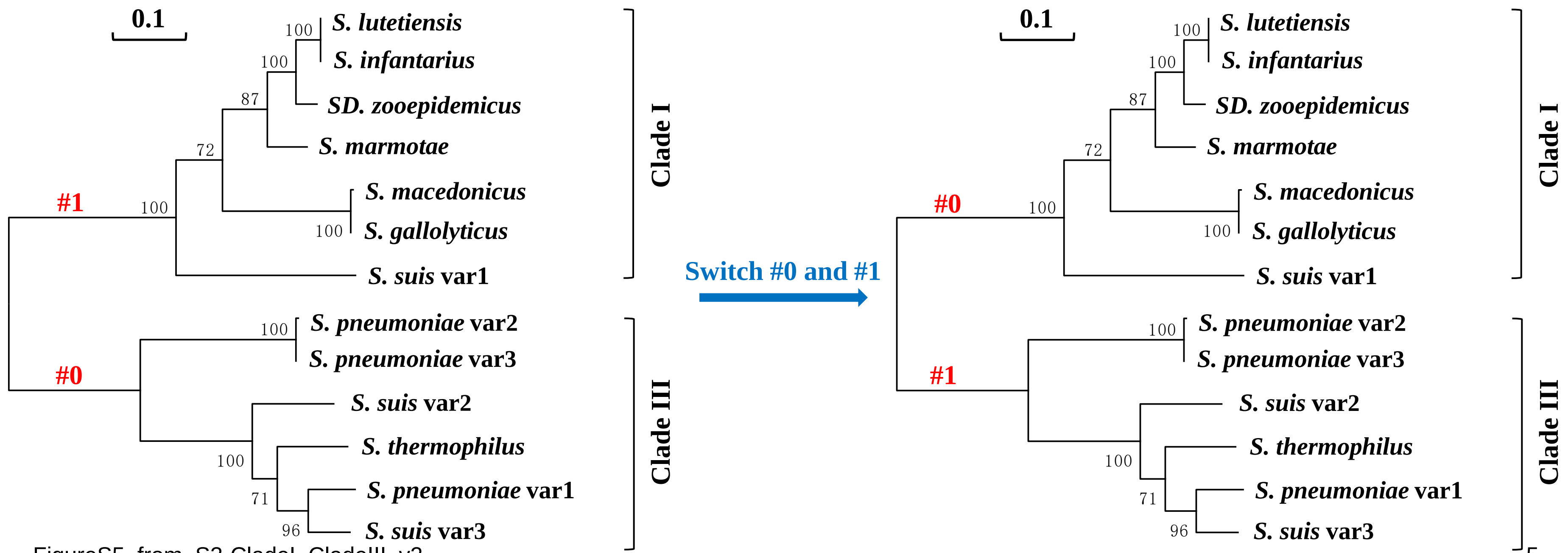

FigureS5_from_S3-CladeI_CladeIII_v2

Table S1. Primers for qRT-PCR assays of IS1239 copies.

Primer	Sequence (5'–>3')	IS1239 location
plr/GAPDH-F	GACGGTACTGAAACAGTTATCTC	1261247
plr/GAPDH-R	GATAGCTTTAGCAGCACCAGTTG	1260237
Loc1-F	CGGATAAGTCTGCTCAAGCTGTC	57870
Loc1-R	GTCGGTTTTCTTGAGGAGCTAGC	59009
Loc4-F	CGGATAAGTCTGCTCAAGCTGTC	505327
Loc4-R	CAACAGATTTAACAGTTGTCATAGC	504317
Loc13-F	CGGATAAGTCTGCTCAAGCTGTC	1490559
Loc13-R	CATACCTTAAAAGTGGCTAAGTTC	1489570
Loc17-F	CGGATAAGTCTGCTCAAGCTGTC	667846
Loc17-R	TGTAGCCAGTGTTACCTTTGCC	668841
Loc21-F	CGGATAAGTCTGCTCAAGCTGTC	1730491
Loc21-R	GTTAAGACACCGCCTTTTCACGG	1731594
Loc22-F	CGGATAAGTCTGCTCAAGCTGTC	1843281
Loc22-R	CCACCTTTTATATGGAAATTAAGC	1842262
htsA-F	GTGTGAATCAGCACCCTAAAACGGC	
htsA-R	CTGGTACATGCCCTCAACACTTCG	
atpB-F	GACCCTACTTGCTGTTTGTATTGTC	
atpB-R	GTCAAAAGCCAGATTGGCTGTTGG	
